# Effect of *H*. *pylori* Infection on Cytokine Profiles and Oxidative Balance in Subjects with Chronic Alcohol Ingestion

**DOI:** 10.1371/journal.pone.0129352

**Published:** 2015-06-18

**Authors:** Baoge Qu, Jiliang Su, Zhongdong Wang, Yafei Wang, Xinghai Han, Hui Wang, Yuanxun Liu, Yiguo Jia, Jindun Pan, Guangying Ren

**Affiliations:** 1 Department of Gastroenterology, Taishan Hospital, Taian, Shandong 271000, P. R. China; 2 Taishan Medical College, Taian, Shandong, 271000, P. R. China; Kermanshah University of Medical Sciences, ISLAMIC REPUBLIC OF IRAN

## Abstract

Different amounts of ingested alcohol can have distinct effects on the human body. However, there is limited research on chronic alcohol consumption with Helicobacter pylori infection. We sought to investigate the relationship between the cytokine profile, oxidative balance and H. pylori infection in subjects with chronic alcohol consumption. A total of 142 subjects were divided into three groups: 59 subjects with chronic alcohol ingestion and H. pylori infection (group A); 53 subjects with chronic alcohol ingestion without H. pylori infection (group B); and 30 control subjects (group C). The serum levels of CagA, interleukin (IL)-10, E-selectin, TNF-α, malondialdehyde (MDA) and superoxide dismutase (SOD) activity were measured by enzyme-linked immunosorbent assay (ELISA). We found that the ages and serum H. pylori CagA levels among the three groups, as well as both the mean drinking age and the mean daily alcohol consumption between groups A and B, were matched and comparable. Comparing the BMIs among the three groups, the BMI differences were found to be statistically significant (F=3.921, P<0.05). Compared with group C, the BMIs in groups A and B were significantly higher (P<0.001 and P<0.01, respectively); however, the BMI differences between group A and group B were not statistically significant (P>0.05). Additionally, no differences in the serum CagA levels were found in comparisons among the groups (all P>0.05). The serum IL-10 and E-selectin levels in group A were significantly lower than those in group B (serum IL-10: P<0.05; E-selectin: P<0.05). The serum IL-10 in group A was significantly higher than that in group C (P<0.01); the serum E-selectin levels in group A did not significantly differ compared with those in group C (P>0.05). Furthermore, the serum IL-10 and E-selectin levels in group B were significantly higher than those in group C (serum IL-10: P<0.001; E-selectin: P<0.05); however, the serum TNF-α levels did not differ among groups (all P>0.05). Although the serum levels of MDA and SOD in groups A and B were slightly lower than those in group C, there were no significant differences among groups (all P>0.05). In conclusion, we believe that H. pylori infection might cause a significant inhibition of certain cytokine profiles in subjects with chronic alcohol ingestion. Moreover, chronically ingested alcohol may exert an adjusted inflammatory effect, but there was no association between H. pylori infection, chronic alcohol consumption and oxidative balance.

## Introduction

Helicobacter pylori (H. pylori) has been recognized as a class I carcinogen by the International Agency for Research on Cancer[1]. Numerous studies have demonstrated that H. pylori infection and alcohol abuse play a role in the pathogenesis of a wide variety of gastrointestinal tract conditions and diseases, including a variety of cancers, and extra-digestive conditions and diseases. H. pylori infection is one of the most common infections in human beings worldwide, infecting up to 50% of the world's population, and it is a human pathogen that colonizes the epithelium of the stomach[2]. Under normal circumstances, the host is not able to clear the infection; thus, it may lead to life-long chronic inflammation[3]. Hence, researchers have paid much attention to H. pylori. Researchers recently[4–6] found that pro- and anti-inflammatory cytokines and oxidative stress play important roles in the development of diseases such as peptic ulceration and gastric adenocarcinoma in patients with H. pylori infection. In contrast, another study regarding the effects of chronic excessive alcohol consumption on the liver and cardiovascular system has demonstrated that modest alcohol intake, such as 1–2 drinks (a standard drink is equivalent to 10 g of ethanol) per day, may decrease cardiovascular mortality[7]. Nonetheless, the mechanisms leading to the various clinical manifestations remain obscure. One study showed that chronic alcohol consumption was associated with enhanced susceptibility to both systemic and mucosal infections, significantly disrupting both peripheral and mucosal immune homeostasis[8]. In addition, drinking and smoking habits may influence the effect of cytokine gene polymorphisms[9]. Furthermore, changes in the cytokine balance are responsible for some of the systemic and hepatic manifestations of alcoholism[10]. Several studies have revealed that both H. pylori infection and chronic alcohol ingestion are important risk factors for some diseases; in particular, they are associated with the occurrence and development of cardiovascular disease, cerebrovascular disease, diabetes mellitus and cancer, thereby posing a severe threat to public health. Animal and human studies have shown that plasma inflammatory reaction-related markers, including interleukin (IL)-10, E-selectin and TNF-α, and oxidative balance markers, including malondialdehyde (MDA) and superoxide dismutase (SOD) activity, play an important role in diseases. In addition, a study showed that cytokines are produced by many cells, and they play the role of mediators in the development of local and systemic inflammatory reactions[11]. Endothelial adhesion molecules (AM) play an important role in the pathogenesis of several diseases, namely, infections, neoplasms and chronic inflammatory diseases[12]. In addition, oxidative stress is suspected to be an early event in the etiology of many disease processes[13]. China has a significantly high prevalence of H. pylori infection while also having high alcohol consumption. Hence, numerous people have suffered from H. pylori infection combined with alcohol intake. The effects of the coexistence of H. pylori infection with alcohol intake and inflammation or lipid peroxidation remain controversial. Therefore, the aims of this study were to evaluate the associations between chronic alcohol ingestion with H. pylori infection and inflammation or lipid peroxidation by measuring the serum levels of inflammatory cytokines, anti-inflammatory cytokines, oxidative stress and antioxidants.

## Materials and Methods

### Subject selection

The study was approved by the clinical research ethics committee of Taishan Hospital of Shandong Province, and all patients provided written informed consent. From January 2012 to December 2013, we prospectively recruited 142 subjects receiving primary care from general healthcare; all subjects received evaluations for chronic alcohol ingestion (diagnosed by a questionnaire regarding alcohol drinking) and H. pylori infection assessments (diagnosed according to a ^13^C-urea breath test [^13^C-UBT]) at Taishan Hospital, Shandong Province. The participants included 112 chronic alcohol ingestion subjects and 30 control subjects. The diagnosis of chronic alcohol ingestion was defined as a daily ethanol intake greater than 40 g in men and 20 g in women for a period longer than 5 years[10]. Additionally, on the same day as the general healthcare examination, the ^13^C-UBT was performed after a fasting period of at least 6 hours with 2 points of collection: baseline and 30 minutes after ingestion of a 75 mg ^13^C-urea capsule diluted in 100 mL of boiled water. The infection status was defined through the analysis of exhaled breath samples by ^13^C infrared spectrometry. A receiver-operating characteristic curve analysis was performed to define the cutoff delta over baseline (DOB) values. DOB≥4 was considered positive, and DOB<4 was considered negative. The patients were divided into three groups: 59 subjects exhibiting H. pylori infection with chronic alcohol ingestion (group A), including 54 males and 5 females with a mean age of 47.03±7.21 years, a mean alcohol drinking history of 5.65±1.61 years, and a mean daily alcohol consumption of 64.27±18.57 g; 53 subjects exhibiting no H. pylori infection with chronic alcohol ingestion (group B), including 49 males and 4 females with a mean age of 46.70±4.62 years, a mean alcohol drinking history of 5.26±1.19 years, and a mean daily alcohol consumption of 62.98±17.80 g; and 30 subjects exhibiting no H. pylori infection without chronic alcohol ingestion (group C), including 27 males and 3 females with a mean age of 45.63±6.28 years. A cross-sectional study was conducted to assess the changes related to inflammation, oxidative stress and antioxidant status in subjects with H. pylori infection with chronic alcohol consumption. In particular, we measured the serum levels of H. pylori cytotoxin-associated gene A (CagA), IL-10, E-selectin, TNF-α, MDA and SOD activity by enzyme-linked immunosorbent assay (ELISA).

Exclusion criteria for the subjects included the following: smoking; fever; infectious diseases; primary and/or secondary gastrointestinal diseases; liver and gallbladder diseases; heart, head, endocrine, nervous, kidney or hematological diseases; electrolyte and acid-base balance disorders; or mental disorders.

### Experimental setup and reagents

A ^13^C infrared spectrometer (Type YH08, Anhui Yanghe Medical Instrument Equipment Co., Ltd.), an Enzyme Standard Instrument (Type ANTHOS 2010, Austria), and a ^13^C-Urea Breath Test Kit (Shenzhen Zhonghe Headway Bio-Sci & Tech Co., Ltd.) were used. H. pylori CagA, IL-10, E-selectin, TNF-α, MDA and SOD kits (Shanghai Enzyme-linked Immune Co. Ltd, made in the United States by R&D) were also used.

### Specimen processing

On the same day as the general healthcare examination, peripheral venous blood samples were collected after overnight fasting for at least 10 hours. For measurements of serum H. pylori CagA, IL-10, E-selectin, MDA and SOD, blood was collected into ice-cold tubes containing EDTA (1 mg/mL), and after centrifugation at 3,000 rpm/minute for 10 minutes, plasma was stored at -70°C until the assay. Repeated freeze-thaw cycles were carefully avoided for all serum samples. According to the manufacturer’s instructions, all parameters were measured by ELISA. The manufacturers’ serum reference levels for all parameters were as follows: H. pylori CagA: 18–700 pg/mL; IL-10: 10–300 ng/L; E-selectin: 2.0–48 ng/L; TNF-α: 20–400 ng/L; MDA: 0.3–8 ng/L; and SOD: 2.5–80 U/mL.

### Statistical analysis

All values were expressed as the mean ± standard deviation. All statistical analyses were performed using the SPSS statistical package (version 19.0 for Windows; SPSS Inc., Chicago). Student’s t-test was used to estimate significant differences between parameters. Among the three groups before statistical analysis, the normal distribution and homogeneity of variances were assessed using the Kolmogorov test. A one-way ANOVA was used to analyze multiple sample means. For multiple post hoc comparisons between groups, variables with a normal distribution were analyzed using the LSD test, and variables without a normal distribution were analyzed using Tamhane’s test. Statistical significance was accepted at P≤0.05.

## Results

A comparison of the ages among the three groups and the mean drinking age, as well as the mean daily alcohol consumption between groups A and B, revealed no significant differences (all P>0.05). Hence, the ages among the three groups, as well as the mean drinking age and mean daily alcohol consumption between groups A and B, were matched and comparable.

Comparison of BMI and CagA among the three groups are listed in Table 1. Comparing the BMIs among the three groups, the BMI differences were found to be statistically significant (F = 3.921, P<0.05). Compared with group C, the BMIs in groups A and B were significantly higher (P<0.001 and P<0.01, respectively); however, the BMI differences between group A and group B were not statistically significant (P>0.05). Additionally, no differences in the serum H. pylori CagA levels were found in comparisons among the three groups (all P>0.05).

**Table 1:** Comparison of BMI and CagA among the three groups (BMI: kg/m^2^, CagA: ng/L, mean ± standard deviation).

*Groups*	*N*	*BMI*	*CagA*
*A*	*59*	*26.18±3.36#*	*78*.*39±55*.*13*
*B*	*53*	*25*.*95±3*.*29*	*85*.*77±62*.*04*
*C*	*30*	*24*.*01±1*.*61*	*61*.*69±32*.*37*
*F*		*3*.*921*	*1*.*907*
*P*		*<0*.*05*	*>0*.*05*

BMI: #P<0.001[Group A vs. Group C]. P<0.01[Group B vs. Group C].

The concentrations of inflammatory markers in the three groups are listed in Table 2. The differences in IL-10 and E-selectin were statistically significant (F = 11.269, P<0.001; F = 5.143, P<0.01, respectively). The difference in TNF-α was not statistically significant (F = 0.760, P>0.05). The serum IL-10 and E-selectin levels in group A were significantly lower than those in group B (serum IL-10: P<0.05; E-selectin: P<0.05). The serum IL-10 in group A was significantly higher than that in group C (P<0.01); the serum E-selectin levels in group A did not significantly differ compared with those in group C (P>0.05). The serum IL-10 and E-selectin levels in group B were significantly higher than those in group C (serum IL-10: P<0.001; E-selectin: P<0.05); however, no differences in the serum TNF-α levels were found in comparisons among the groups (all P>0.05).

**Table 2:** Comparison of serum IL-10, E-selectin and TNF-α levels among the three groups (ng/L, mean ± standard deviation).

*Groups*	*N*	*IL-10*	*E-selectin*	*TNF-α*
*A*	*59*	*402*.*69±273*.*44* †	*31*.*93±25*.*38* *	*274*.*60±257*.*00*
*B*	*53*	*553*.*79±354*.*29* †† §	*46*.*45±34*.*93* **	*319*.*52±242*.*56*
*C*	*30*	*244*.*03±165*.*94*	*29*.*34±16*.*03*	*264*.*52±117*.*22*
*F*		*11*.*269*	*5*.*143*	*0*.*760*
*P*		*<0*.*001*	*<0*.*01*	*>0*.*05*

IL-10:

^†^ P<0.05[Group A vs. Group B].

^††^P<0.01[Group A vs. Group C].

^§^P<0.001[Group B vs. Group C].

E-selectin:

* P<0.05[Group A vs. Group B].

** P<0.05[Group B vs. Group C].

The concentrations of the markers of lipid peroxidation and antioxidant status are listed in Table 3. The differences in MDA and SOD were not statistically significant (F = 2.605, P>0.05; F = 1.612, P>0.05, respectively). Although the serum levels of MDA and SOD in groups A and B were slightly lower than those in group C, there were no significant differences among groups (all P>0.05).

**Table 3:** Comparison of serum MDA and SOD levels among the three groups (MDA: ng/L, SOD: U/mL, mean ± standard deviation).

*Groups*	*N*	*MDA*	*SOD*
*A*	*59*	*6*.*71±6*.*41*	*65*.*58±63*.*29*
*B*	*53*	*9*.*47±9*.*05*	*89*.*68±78*.*08*
*C*	*30*	*10*.*52±10*.*01*	*83*.*72±81*.*87*
*F*		*2*.*605*	*1*.*612*
*P*		*>0*.*05*	*>0*.*05*

## Discussion

H. pylori colonizes the stomach and induces strong mucosal inflammation and a local and systemic immune response. However, we were unable to draw a consistent conclusion about the associations between H. pylori and both systemic inflammation and the immune response. One study[14] revealed that H. pylori might stimulate and induce the upregulation of E-selectin, IL-8 and IL-6. Another comparative study[2] showed that induced H. pylori-related TNF-α is concentrated in the gastric mucosa; however, this pathogen does not cause any significant changes in the gastric mucosa level of IL-10 and the serum levels of TNF-α and IL-10. Similarly, H. pylori infection in children does not cause changes in systemic cytokine secretion (including IL-1, IL-1β, IL-6, IL-8, TNF-α, and IFN-γ)[15]. Conversely, the gene expression levels of IL-10 messenger RNA (mRNA) were significantly higher in the H. pylori(+) (Hp[+]) gastritis group than in the control group (P<0.01)[16]. In addition, high IL-10 expression may indicate that regulatory T cells also play a role in the chronic phase of H. pylori infection[17]. Moreover, upregulated E-selectin expression was found to be localized to the gastric mucosa rather than being a systemic response to the infection[2]. Furthermore, a study of mice deficient in IL-10[18] suggested that endogenous IL-10 inhibits the protective immune response to H. pylori infection. CagA-positive H. pylori infection plays a critical role in various diseases via changes in inflammation and immune response. The data from one study[19] demonstrated that a CagA+ strain of H. pylori not only can be a trigger but also may result in chronic inflammation involved in the pathogenesis of cardiac syndrome X (CSX). In addition, the cag pathogenicity island (cagPAI) is critical for H. pylori pathogenesis in bile duct cells[20]. The pathogenic mechanism of CagA-positive H. pylori infections demonstrates that CagA is crucial for dendritic cell tolerization through the modulation of IL-10 secretion[21]. High levels of IL-18 in CagA-positive subjects predispose them to susceptibility to digestive ulcers[22], and the virulent H. pylori strains may cause inflammation by stimulating epithelial cells through cag-encoded proteins and mononuclear inflammatory cells through duodenal ulcer-promoting gene A1 products[23]. Hence, H. pylori infection may result in certain changes in cytokines. Meanwhile, alcohol consumption is associated with cytokine alterations and has been shown to be responsible for some diseases. Alcohol consumption has an association with alterations in TNF-α and IL-6 serum levels[24] and dysregulated cytokine levels (IL-10, IL-12 and IFN-γ)[25], and moderate drinkers also exhibit higher serum levels of ICAM-1 and E-selectin than those observed in abstainers[26]. Additionally, chronic inflammation associated with obesity may play a role in the etiology of several diseases[27]. One study showed that BMI was the only predictor of hs-CRP levels and TNF-α levels (all P<0.001), considering that obesity induced an increase in certain inflammatory markers in the serum[28]. Our present study revealed that BMI and some inflammatory and anti-inflammatory markers were significantly increased in subjects with chronic alcohol ingestion without H. pylori infection compared with the control group, suggesting that chronically ingested alcohol may cause overweight and exert a modulatory effect on the inflammatory response. Hence, we deduced that the changes in chronically ingested alcohol-related cytokine profiles are associated with chronically ingested alcohol per se and that a high quantity of calories due to chronically ingested alcohol causes overweight or obesity. There are few studies of H. pylori infection with chronically ingested alcohol, and the role of H. pylori infection in the metabolism of ingested ethanol has not been fully elucidated. One study[29] showed that BMI was significantly decreased six months after the eradication of H. pylori, and the eradication of H. pylori had an impact on BMI. Our study found that BMI and the inflammatory response, as indicated by E-selectin levels, and the anti-inflammatory response, as reflected by the IL-10 levels, were significantly increased in subjects with H. pylori infection with chronic alcoholic ingestion compared with the control group subjects and were significantly reduced in chronic alcoholics with H. pylori infection compared with patients with chronic alcohol ingestion and no H. pylori infection. However, no differences in BMI, serum CagA or TNF-α levels were found in comparisons between chronic alcoholics with H. pylori infection and chronic alcoholics without H. pylori infection, suggesting that H. pylori infection might result in a significant inhibition of some cytokine profiles in subjects with chronic alcohol ingestion. Not all of the results of the present study were in accordance with the previous literature, which is considered to result from the interaction between H. pylori infection and chronically ingested alcohol. Hence, its putative mechanism is interactions between H. pylori infection and the metabolism of ingested ethanol caused by H. pylori infection, resulting in significantly decreased class I[30] and IV alcohol dehydrogenase[30,31] activities. In addition, our finding that H. pylori infection with ingested ethanol correlates with BMI and our conclusion, which demonstrated that the metabolism of small amounts of ethanol is attenuated in subjects with H. pylori infection[32], seem to support the above opinion. However, because the serum H. pylori CagA levels among the three groups were not different, it is necessary to conduct further research to determine the relationship between H. pylori CagA and cytokine profiles.

H. pylori- and/or alcohol-induced oxidative stress appear to be linked to H. pylori infection and the metabolism of ethanol, but this is not a definitive conclusion. Our present study revealed that the oxidative stress levels were not significantly changed among the groups, suggesting that oxidative balance had no association with H. pylori infection and/or chronic alcohol ingestion. In contrast, an increased level of oxidative stress was also found in H. pylori-infected individuals[33]. Reactive oxygen species (ROS) production was increased in peripheral blood by H. pylori infection[34]. Oxidative stress plays a role in tissue damage in H. pylori infection[35]. H. pylori may be one of the causes of high MDA levels[36]. Additionally, there is a close relationship between plasma MDA and nitric oxide levels and the gastric histopathology and genotypes of H. pylori[6]. In contrast, another study revealed that H. pylori infection did not have significant effects on oxygen metabolism in the inhabitants of Łódź[37]. Lipid peroxidation is a mechanism involved in the pathogenesis of gastritis, whether associated or not associated with H. pylori infection[38]. However, the H. pylori CagA gene is relevant to the expression of antioxidant proteins of H. pylori[39]. In addition, total SOD activity increased after exposure to CagA(+) strains and was marginally increased with exposure to CagA(-) strains[40]. Studies have shown that ethanol consumption may cause increased oxidative stress with increased formation of lipid peroxides and free radicals. Acetaldehyde is an oxidative stress inducer via injured mitochondria[41]. Oxidative stress resulting from ethanol oxidation is one established pathogenic event in alcohol-induced toxicity[6]. Additionally, binge ethanol administration significantly enhanced endogenous lipid peroxidation and caused enhanced in vitro susceptibility to lipid peroxidation. SOD activity was found to be significantly increased[42]. However, our study showed that there was no association between H. pylori infection, chronic alcohol consumption and oxidative balance. This finding does not support the previous conclusions about H. pylori-, H. pylori CagA- and/or alcohol-induced oxidative stress. Hence, it will be necessary to confirm these associations with large case studies.

In conclusion, chronic alcohol ingestion with or without H. pylori infection might elevate BMI and certain serum cytokine markers (i.e., E-selectin and IL-10, but not TNF-a). Moreover, H. pylori infection may result in significantly reduced levels of pro- and anti-inflammatory cytokines in subjects with chronic alcohol consumption; however, changes in serum H. pylori CagA and oxidative stress were not obvious. Hence, chronic alcohol ingestion with or without H. pylori infection may partly change the BMI and cytokine profile but not the H. pylori CagA and oxidative balance. Thus, it will be necessary to determine the exact mechanism in a future study.

Our study has several limitations. First, the subject study sample was small and biased by a disproportionate male predominance. Second, we did not control for the potential impact of other factors, such as drugs and co-morbidities, including steatosis, steatohepatitis, and obesity (all potentially influencing the studied variables). Third, our conclusions are based on a prospective observational study rather than a completely randomized controlled study. Fourth, data on the roles of different types and amounts of alcohol in subjects with H. pylori were not collected. Finally, the levels of other well-characterized cytokines, such as IFN-γ, were not examined in our study.
